# Myocardial Injury in COVID-19 Patients: Association with Inflammation, Coagulopathy and In-Hospital Prognosis

**DOI:** 10.3390/jcm10102096

**Published:** 2021-05-13

**Authors:** Victor Arévalos, Luis Ortega-Paz, Juan José Rodríguez-Arias, Margarita Calvo, Leticia Castrillo, Anthony Salazar, Merce Roque, Ana Paula Dantas, Manel Sabaté, Salvatore Brugaletta

**Affiliations:** 1 Department of Cardiology, Clinic Cardiovascular Institute, Hospital Clinic, 08036 Barcelona, Spain; varevalos88@gmail.com (V.A.); lgortega@clinic.cat (L.O.-P.); juanjose.rodriguez.a@gmail.com (J.J.R.-A.); mcalvol@clinic.cat (M.C.); castrillo@clinic.cat (L.C.); aesalazar@clinic.cat (A.S.); mroque@clinic.cat (M.R.); masabate@clinic.cat (M.S.); 2 Institut d’Investigacions Biomèdiques August Pi i Sunyer (IDIBAPS), University of Barcelona, 08036 Barcelona, Spain; adantas@clinic.cat

**Keywords:** myocardial injury, coagulopathy, mortality, coronavirus disease 2019

## Abstract

The exact mechanisms leading to myocardial injury in the coronavirus disease 2019 (COVID-19) are still unknown. In this retrospective observational study, we include all consecutive COVID-19 patients admitted to our center. They were divided into two groups according to the presence of myocardial injury. Clinical variables, Charlson Comorbidity Index (CCI), C-reactive protein (CRP), CAC (COVID-19-associated coagulopathy), defined according to the ISTH score, treatment and in-hospital events were collected. Between March and April 2020, 331 COVID-19 patients were enrolled, 72 of them (21.8%) with myocardial injury. Patients with myocardial injury showed a higher CCI score (median (interquartile range), 5 (4–7) vs. 2 (1–4), *p* = 0.001), higher CRP values (18.3 (9.6–25.9) mg/dL vs. 12.0 (5.4–19.4) mg/dL, *p* ˂ 0.001) and CAC score (1 (0–2) vs. 0 (0–1), *p* = 0.001), and had lower use of any anticoagulant (57 patients (82.6%) vs. 229 patients (90.9%), *p* = 0.078), than those without. In the adjusted logistic regression, CRP, myocardial injury, CCI and CAC score were positive independent predictors of mortality, whereas anticoagulants resulted as a protective factor. Myocardial injury in COVID-19 patients is associated with inflammation and coagulopathy, resulting in a worse in-hospital prognosis. Treatment with anticoagulant agents may help to improve in-hospital outcomes.

## 1. Introduction

Coronavirus disease 2019 (COVID-19) is an infectious disease caused by the severe acute respiratory syndrome coronavirus-2 (SARS-CoV-2), representing a significant threat to health worldwide [1]. The fast spread of this disease at pandemic level has overloaded hospitals, generating important efforts to understand its pathophysiology in order to improve its prognosis. Although the primary target of the SARS-CoV-2 is the respiratory system, it could potentially affect many other systems [2]. The relationship between COVID-19 and the cardiovascular system is currently under investigation. It is known that a certain degree of myocardial injury, defined as blood elevated levels of cardiac biomarkers (e.g., Troponin I), can be present and that it may be related to the severity of the disease [3,4,5,6,7]. However, the mechanism of myocardial injury caused by COVID-19 is unknown.

The main objective of this study was to investigate the association between the myocardial injury, inflammation and coagulopathy in COVID-19 patients, analyzing in particular its impact on in-hospital mortality.

## 2. Materials and Methods

### 2.1. Study Design and Participants

This is a retrospective registry of all the consecutive subjects tested for COVID-19 disease in the University Hospital Clínic de Barcelona, a tertiary referral hospital affiliate to the University of Barcelona. In this specific study, we included all patients with a laboratory-confirmed SARS-CoV-2 infection (polymerase chain reaction test of respiratory samples) who had a troponin determination during hospitalization. The high-sensitivity cardiac troponin I (hs-cTnI) measurement determination was performed under the clinical judgment of each treating physician. However, the employed clinical protocol in that period recommended the measurement of hs-cTnI for patient prognosis stratification at the patient admission. This retrospective study was conducted from 15 March to 30 April 2020. This study was approved by the Ethics Committee of our Institution and complies with local regulations and internationally established principles of the Declaration of Helsinki. Given the registry’s anonymous characteristics and the health alarm situation generated by the COVID-19 pandemic, written informed consent was waived.

### 2.2. Data Collection

The following variables were collected: demographic baseline characteristics, COVID-19 symptoms, comorbidities, laboratory findings during hospitalization (including cardiac biomarkers highest values, C-reactive protein (CRP) highest value, D-dimer highest value, prothrombin time (PT) highest value in seconds, platelets lowest value and fibrinogen lowest value), intensive care unit admission (ICU), treatment (drugs including vasoactive agents and anticoagulation, invasive mechanical ventilation), in-hospital complications and outcome. All data were independently reviewed and entered into a computerized database (V.A. and J.J.R.). Patients were classified according to myocardial injury, defined as blood levels of high-sensitivity cardiac troponin I (hs-cTnI) above the 99th percentile upper reference limit according to the fourth universal definition of myocardial infarction of 2018 [8]. Hs-cTnI levels have been measured using Siemens Healthineers high-sensitivity troponin I assay.

Charlson Comorbidity Index (CCI) was used to assess patient’s comorbidities. CCI is a validated index for predicting life expectancy at ten years, depending on the age at which it is evaluated and on the subject’s comorbidities [9].

Regarding in-hospital complications, we recorded the following: acute coronary syndrome (ACS), stroke, deep venous thrombosis (DVP), pulmonary embolism (EP), major bleeding, red blood cell transfusion and serious cardiac arrhythmias.

### 2.3. Definitions

COVID-19-associated coagulopathy (CAC) was assessed using International Society on Thrombosis and Hemostasis (ISTH) interim guidance for recognition and management of CAC, which allows to identify patients with coagulation activation and to estimate the severity of coagulopathy. This scoring system considers the following variables:

1. Prolonged PT at least 25%,
2. Platelets count lower than 100 × 109/L,
3. Fibrinogen lower than 2 g/dL,
4. Increased D-dimer more than four times the normal value (500 ng/mL) [10].

The score can go from zero, in a patient without any of these features, up to four, when all the above-mentioned are met.

The anticoagulant treatment included unfractionated heparin (UFH), low-molecular-weight heparin (LMWH), direct oral anticoagulants (DOACs) or vitamin K antagonists (VKA). If the patient received at least one dose of anticoagulant treatment, they were included in the anticoagulant treatment group. Patients were categorized as being on prophylactic anticoagulation if on subcutaneous UFH, or LMWH once daily, or apixaban (2.5 mg twice a day or 5 mg daily in patients ≤ 75 years) were administered. The anticoagulation therapeutic regimen was defined as one of the following: continuous intravenous infusions of UFH, high-dose LMWH (specifically enoxaparin 1 mg/kg twice daily or 1.5 mg/kg daily), apixaban 5 mg twice daily, rivaroxaban or dabigatran. For patients > 75 years, apixaban was considered therapeutic at lower doses: at 2.5 mg twice a day or 5 mg once a day.

Myocardial infarction was defined according to the fourth universal definition [8]. Major bleeding was defined as a type 3 of the Bleeding Academic Research Consortium (BARC) or higher [11]. Serious cardiac arrhythmias were defined as: bradycardia requiring intravenous medication or pacemaker, supraventricular tachycardia requiring intravenous medication or cardioversion, or ventricular tachycardia requiring intravenous medication or cardioversion.

### 2.4. Statistical Analysis

Continuous variables are presented as mean ± standard deviation (SD), or as median and interquartile range (IQR), as appropriate. Categorical variables are reported as absolute numbers and percentages. Differences in percentages of categorical variables were compared between groups with Chi-square test or Fisher’s exact test. Differences in continuous variables were compared with a Student’s *t*-test or Mann–Whitney U test, as appropriate. Two logistic regression analyses were performed to determine the predictors of myocardial injury and in-hospital all-cause mortality. In the first, the following variables were entered: sex, CCI score, CRP highest blood level, CAC score and any in-hospital anticoagulant treatment; in the second, the same variables as above and presence of myocardial injury were entered. These variables were used because they have been associated with prognosis of COVID-19 patients in previous studies [5,10,12,13,14,15,16].

All *p*-values reported are two-sided, and a two-sided probability value < 0.05 was considered statistically significant. All data were processed using the Statistical Package for Social Sciences, version 25.0 (SPSS Inc., Chicago, IL, USA).

## 3. Results

### 3.1. Baseline Characteristics

**Figure 1:**
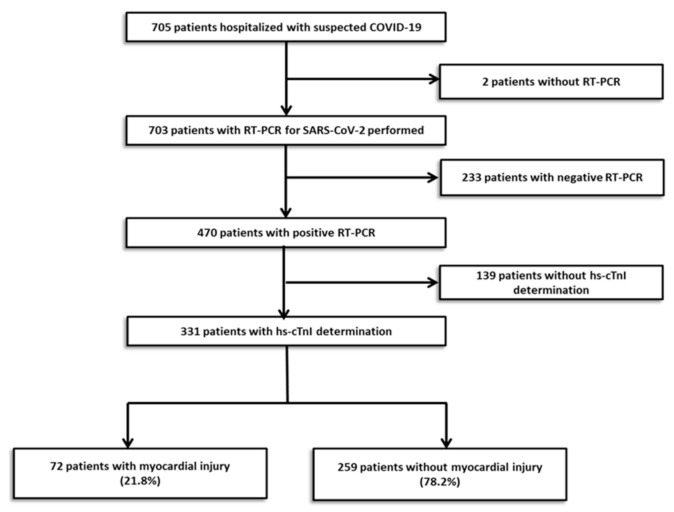
Flowchart of participant selection.

A total of 705 subjects were tested for COVID-19 from 15 March to 30 April 2020. Results of the PCR test were missing in 2 patients and were negative in 233. Out of the positive patients, only 331 have a determination of hs-cTnI during hospitalization and they were eventually entered in this analysis. Myocardial injury, defined as at least one value of hs-cTnI above the 99th percentile upper reference limit, was detected in 72 patients (21.8%) (Figure 1). The median (IQR) time from the beginning of symptoms to Hs-cTnI peak was 7.0 (3.0–13.0) days in the patients with myocardial injury.

Patients with myocardial injury were older, more frequently male and with higher incidence of hypertension, diabetes, cardiovascular disease and a higher CCI score compared with patients without (Table 1). Baseline medication and symptoms are shown in Supplementary Tables S1 and S2, respectively. More than a half of the patients (56.2%) referred dyspnea before hospital admission.

In particular, during hospitalization, a larger number of patients without myocardial injury were under anticoagulant treatment than those with (88.4% vs. 79.2%, *p* = 0.051). In-hospital treatment, including cardiovascular medication and specific drugs used in COVID-19 treatment, are listed in Supplementary Table S3.

### 3.2. Inflammation and Coagulopathy

CRP was higher in patients with myocardial injury (18.3 (9.6–25.9) mg/dL vs. 12.0 (5.4–19.4), *p* = 0.001) than in those without. Patients with myocardial injury had lower levels of hemoglobin and lower counts of lymphocytes and platelets, compared with patients without. Moreover, they also had higher values of D-dimer (median (IQR) mg/dL, 4250 (1500–8600) mg/dL vs. 1700 (800–4300) mg/dL) and longer PT (14.7 (13.4–17.3) seconds vs. 13.1 (12.3–14.0) seconds) as compared to those without. The peak creatinine levels were significantly higher in patients with myocardial injury compared to those without (2.02 mg/dL (1.32–3.69) vs. 0.95 mg/dL (0.77–1.19); *p* = 0.001). Besides, NT-proBNP levels were also significantly higher in patients with myocardial injury (Table 2).

**Figure 2:**
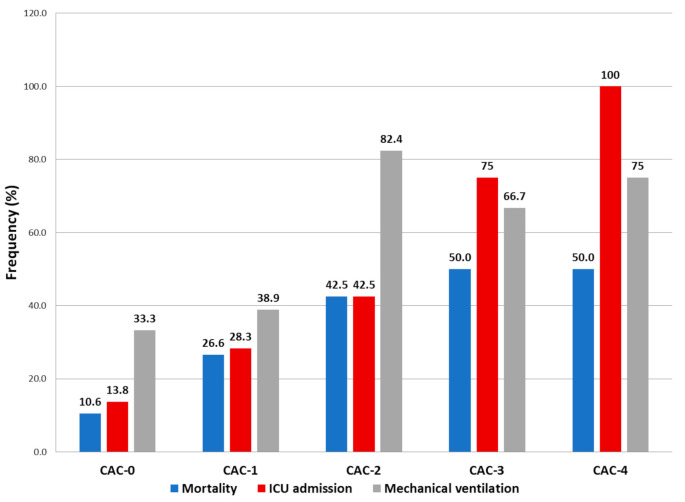
Incidence of mortality, ICU (intensive care unit) admission and mechanical ventilation in patients grouped according to their CAC (COVID-19-associated coagulopathy) score. Higher CAC scores were associated to higher ICU admission rates, higher invasive mechanical ventilation and higher mortality.

The CAC score was higher in patients with myocardial injury (1 (0–2) vs. 0 (0–1), *p* = 0.001), with at least one parameter of CAC in 72.2% of the patients with myocardial injury vs. 49.4% of the patients without. A higher CAC score was associated with higher rates of ICU admission, mechanical ventilation and all-cause mortality (Figure 2).

Independent predictors of myocardial injury at logistic regression analysis were CCI score (OR: 1.550, CI: 1.363–1762, *p* ˂ 0.001), CRP (OR: 1.059, CI: 1.023–1.097, *p* = 0.001) and CAC score (OR: 2.085, CI: 1.447–3.005, *p* ˂ 0.001), while the treatment with anticoagulants resulted as a protective factor (OR: 0.204, CI: 0.077–0.540, *p* = 0.001).

### 3.3. In-Hospital Outcomes

Overall, patients with myocardial injury showed higher incidence of in-hospital events and higher all-cause mortality (48 (66.7%) vs. 25 (9.7%), *p* ˂ 0.001) as compared to patients without (Table 3).

During the in-hospital stay, 10 patients in the group of myocardial injury developed an ACS (6 of them had myocardial injury before the ischemic event), 2 ST-elevation myocardial infarction (STEMI) cases and 8 non-ST-elevation myocardial infarction (NSTEMI). Both cases of STEMI underwent thrombolysis with inadequate response and death before catheterization (Supplementary Table S4).

At logistic analysis, CCI score (OR: 1.469, CI: 1.265–1.705, *p* ˂ 0.001), CRP (OR: 1.094, CI: 1.051–1.139, *p* ˂ 0.001), CAC score (OR: 1.589, CI: 1.056–2.390, *p* = 0.026) and myocardial injury (OR: 6.398, CI: 2.999–13.649, *p* ˂ 0.001) were independent predictors of in-hospital mortality, whereas therapy with anticoagulants resulted as a protective factor (OR: 0.152, CI: 0.05–0.458, *p* = 0.001).

## 4. Discussion

The main findings of our analysis were: (1) myocardial injury is present in up to 21.8% of patients with COVID-19 admitted to the hospital, (2) it is associated with activation of inflammation and coagulopathy, with a higher in-hospital mortality, and (3) anticoagulant treatment may be related with lower incidence of myocardial injury and mortality.

In patients with COVID-19, myocardial injury is part of the currently known multi-system affectation, and it has demonstrated an impact on the prognosis [4,5,6,7,17]. The incidence of myocardial injury varies between studies, depending on the sample and characteristics of the included patients. A review of 26 studies including 11,685 patients showed a weighted pooled prevalence of myocardial injury of 20% (ranged from 5% to 38% depending on the criteria used in the analysis) [7], which is in line with our analysis. In our study, the excluded patients (*n* = 139, because of absence of hs-cTnI determination) were probably considered at very low risk of adverse events by their treating physician. Ultimately, they exhibited a good prognosis with a mortality rate of 2.9%.

The pathophysiology and pathways that conduct to the myocardial injury in COVID-19 patients are still unknown. One of the proposed mechanisms is related to the angiotensin-converting enzyme (ACE) 2 receptor, which has been postulated as the host cellular receptor for virus spike (S) protein. The ACE2 receptors are highly expressed in adult human hearts and endothelial cells, indicating an intrinsic susceptibility of heart and blood vessels to a direct invasion of SARS-CoV-2 [18,19]. In our analysis, we have found interplay between myocardial injury, inflammation and thrombosis, which may help to explain the cause of myocardial injury.

It is known that inflammation is an essential mechanism in the pathophysiology of COVID-19, and that an exacerbated inflammatory response could lead to pulmonary and systemic consequences. This inflammatory environment is evidenced by elevated biomarkers such as CRP, ferritin and interleukin 6 (IL-6) [20,21]. We found in our study that higher levels of CRP are significantly related to worse progression of the disease, including higher troponin levels, myocardial injury development and all-cause mortality.

Inflammation is also closely related to the development of coagulopathy and a resultant pro-thrombotic state. The activation of thrombosis due to inflammation has many pathways, including: a higher expression of tissue factor in response to IL-6, excessive complement activation or neutrophil extracellular traps (NETs) [22]. The pro-thrombotic state with generation of thrombi in the macro- and micro-circulation is another potential cause of myocardial injury in COVID-19 patients [7,19,23]. All of these coagulation disturbances were described under the denomination of CAC.

In the context of CAC, increased D-dimer and prolonged PT have been more frequently found in critically ill COVID-19 patients and represent a marker of poor prognosis [10,24,25,26]. In addition to these prior observations, we provide an interesting relationship between CAC and myocardial injury. CAC score was indeed an independent predictor of myocardial injury and subsequent worse prognosis. All of these findings may strongly support a key role of coagulopathy in the COVID-19 cardiac involvement.

Given all this background, treatment of coagulopathy may be beneficial for these patients. Available evidence from observational studies has shown that the use of heparin, LMWH or UFH, with prophylactic or therapeutic dose, was associated with lower mortality in COVID-19 patients [27,28]. A recent randomized phase II clinical trial contributes to this evidence, showing an improvement in the gas exchange, reduced D-dimer levels and a higher ratio of successful weaning from mechanical ventilation after respiratory failure with a therapeutic dose of heparin rather than a prophylactic dose [29].

In our study, we indeed found a protective value of any anticoagulant treatment in terms of all-cause death. LMWH was the main anticoagulant treatment used, and it may have prevented micro-thrombi generation in the cardiac and pulmonary circulation. Of note, it is also the anti-inflammatory property of the LMWH [29,30]. Heparin has also been implicated in binding to COVID-19 S proteins as well as downregulating IL-6 [31]. The potential benefit of anticoagulation treatment is hypothesis-generating, and it should be studied in controlled and randomized trials with a larger number of patients to demonstrate a real effect in prognosis.

## 5. Limitations

This study has some limitations that should be acknowledged. First, it is a retrospective study based on the review of medical reports, and there could be a selection bias because biomarkers were not measured in all patients, but in some based on the criterion of the treating physician. Second, echocardiographic evaluation was performed only in the highly selected cases (4.2%) during hospitalization and precluded to relate the biomarkers findings with potential impairments of ventricular function. Nevertheless, due to the center’s policies, complementary tests were performed only in selected cases to reduce the risk of healthcare professional exposition. Third, due the several differences in baseline characteristics, the effect of persistent confounders cannot be ruled out. Fourth, the timing relationship between the hs-cTnI peak and the start of anticoagulation was not assessed.

## 6. Conclusions

Myocardial injury is a frequent finding in hospitalized COVID-19 patients, and it is associated with the presence of higher levels of inflammatory and coagulopathy biomarkers, being a strong independent predictor of in-hospital mortality. Treatment with anticoagulant agents, using prophylactic or therapeutic doses, may help to reduce myocardial injury and in-hospital mortality.

## Figures and Tables

**Table 1:** Baseline characteristics. In-hospital anticoagulation.

	Myocardial Injury		*p*-Value
	With (*n* = 72)	Without (*n* = 259)	
Characteristic			
Age (years), mean ± SD	75 ± 11	61 ± 17	0.001
Male sex, *n* (%)	50 (69.4)	145 (56.0)	0.043
BMI, median (IQR)	28.6 (25.8–30.4)	27.0 (24.2–29.8)	0.071
Current smoker, *n* (%)	2 (2.8)	7 (2.7)	1.000
Diabetes, *n* (%)	28 (38.9)	39 (15.1)	0.001
Hypertension, *n* (%)	54 (75.0)	107 (41.3)	0.001
Hypercholesterolemia, *n* (%)	33 (45.8)	70 (27.0)	0.004
Chronic kidney disease, *n* (%)	34 (47.2)	35 (13.5)	0.001
Hemodialysis, *n* (%)	4 (5.6)	2 (0.8)	0.022
Atrial fibrillation, *n* (%)	7 (9.7)	13 (5.0)	0.161
Previous stroke or TIA, *n* (%)	10 (13.9)	6 (2.3)	0.001
Previous AMI, *n* (%)	11 (15.3)	8 (3.1)	0.001
Previous PCI, *n* (%)	14 (19.4)	10 (3.9)	0.001
Previous CABG, *n* (%)	4 (5.6)	2 (0.8)	0.022
Previous PVD, *n* (%)	5 (16.7)	2 (2.2)	0.001
Previous COPD or Asthma, *n* (%)	11 (15.3)	32 (12.4)	0.553
Previous pneumonia, *n* (%)	3 (4.2)	3 (3.5)	0.732
Previous heart failure, *n* (%)	12 (16.7)	10 (3.9)	0.001
Previous bleeding	9 (12.5)	9 (3.5)	0.006
LVEF, mean ± SD	53 ± 9	57 ± 8	0.106
Previous PE, *n* (%)	2 (2.8)	3 (1.2)	0.299
Active cancer, *n* (%)	10 (13.9)	12 (4.6)	0.013
Organ transplant, *n* (%)	3 (4.2)	9 (3.5)	0.728
Charlson Comorbidity Index, median (IQR)	5 (4–7)	2 (1–4)	0.001
Anticoagulation			
Any anticoagulants, *n* (%)	57 (79.2)	229 (88.4)	0.051
LMWH	58 (81.0)	225 (87.0)	0.178
DOAC	0	4 (1.5)	0.580
Prophylactic indication	46 (80.7)	203 (88.6)	0.001

AMI, acute myocardial infarction; BMI, body mass index; CABG, coronary artery bypass graft; COPD, chronic obstructive pulmonary disease; DOAC, direct-acting oral anticoagulants; IQR, interquartile range; LMWH, low-molecular-weight heparin; LVEF, left ventricular ejection fraction; PCI, percutaneous coronary intervention; PE, pulmonary embolism; PVD, peripheral vascular disease I; SD, standard deviation; TIA, transient ischemic accident.

**Table 2:** Biomarkers.

Biomarkers	Myocardial Injury		*p*-Value
	With (*n* = 72)	Without (*n* = 259)	
NT-ProBNP (pg/mL), Median (IQR) ^a^	3009 (1288–4523)	409 (156 –1486)	0.001
Hemoglobin (g/L), Median (IQR) ^b^	105 (84 –119)	121 (109 –134)	0.001
Creatinine (mg/dL), Median (IQR) ^a^	2.02 (1.32–3.69)	0.95 (0.77–1.19)	0.001
Lymphocytes (109/L), Median (IQR) ^b^	0.3 (0.2–0.5)	0.65 (0.40–1.0)	0.001
CRP (mg/dL), Median (IQR) ^a^	18.3 (9.6–25.9)	12.0 (5.4–19.4)	0.001
Creatinine (mg/dL), Median (IQR) _a_	2.02 (1.32–3.69)	0.95 (0.77–1.19)	0.001
Thrombotic biomarkers			
D-dimer (ng/mL), Median (IQR) ^a^	4250 (1500–8600)	1700 (800–4300)	0.001
PT (segundos), Median (IQR) ^a^	14.7 (13.4–17.3)	13.1 (12.3–14.0)	0.001
Platelets (109/L), Median (IQR) ^b^	138 (95–189)	171 (134–215)	0.001
Fibrinogen (g/L), Median (IQR) ^b^	3.40 (2.4–6.0)	4.0 (2.8–5.8)	0.512
CAC			
CAC score, median (IQR)	1 (0–2)	0 (0–1)	0.001
CAC 0 feature, *n*(%)	20 (27.8)	131 (50.6)	0.001
CAC 1 feature, *n*(%)	25 (34.7)	103 (39.8)	
CAC 2 feature, *n*(%)	19 (26.4)	21 (8.1)	
CAC 3 feature, *n*(%)	5 (6.9)	3 (1.2)	
CAC 4 feature, *n*(%)	3 (4.2)	1 (0.4)	

CAC, COVID-19-associated coagulopathy; CRP, C-reactive protein; hs-cTnI, high-sensitivity troponin; PT, prothrombin time. a corresponds to the highest value recorded during admission. b corresponds to the lowest value recorded during admission. Hs-cTnI, high-sensitivity cardiac Troponin I; RT-PCR, reverse transcription polymerase chain reaction.

**Table 3:** In-hospital days, ICU treatment and outcomes.

	Myocardial Injury		*p*-Value
	With (*n* = 72)	Without (*n* = 259)	
Hospitalization days, mean ± SD	15.3 ± 12.9	15.85 ± 9.0	0.751
Need for ICU, *n* (%)	25 (34.7)	58 (22.4)	0.046
UCI days, mean ± SD	12.5 ± 9.6	7.9 ± 5.3	0.032
Invasive mechanical ventilation, *n* (%)	21 (29.1)	21 (8.1)	0.001
Need for vasoactive drugs, *n* (%)	20 (27.8)	29 (11.2)	0.001
Need for IMV, *n* (%)	4 (5.6)	5 (1.9)	0.107
ACS, *n* (%)	10 (13.9)	0	0.001
NSTEMI	8 (11.1)	0	
STEMI	2 (2.8)	0	
Stroke, *n* (%)	0 (0)	1 (0.4)	1.000
Ischemic	0	1 (0.4)	
DVT, *n* (%)	2 (2.8)	2 (0.8)	0.207
PE, *n* (%)	3 (4.2)	14 (5.4)	1.000
Bleeding, *n* (%)	8 (11.1)	3 (1.2)	0.001
Red blood cell transfusion, *n* (%) *	18 (25)	6 (2.3)	0.001
Cardiac arrhythmias, *n* (%) **	8 (11.1)	0 (0)	0.001
All-cause mortality, *n* (%)	48 (66.7)	25 (9.7)	0.001

ACS, acute coronary syndrome; DVT, deep-vein thrombosis; IMV, invasive mechanical ventilation; NSTEMI, non-ST elevation myocardial infarction; PE, pulmonary embolism; STEMI, ST elevation myocardial infarction. * Need for transfusion of red blood cell concentrates. ** All cases corresponded to atrial fibrillation or supraventricular tachycardia with need for intravenous medication.

## Data Availability

The data presented in this study are available upon request from the corresponding author.

## Supplementary Materials

The following are available online at https://www.mdpi.com/article/10.3390/jcm10102096/s1, Table S1: Baseline medication; Table S2: Symptoms; Table S3: In-hospital treatment; Table S4: Myocardial infarction during hospitalization.

## References

[1] Yang C.L., Qiu X., Zeng Y.K., Jiang M., Fan H.R., Zhang Z.M. (2020). Coronavirus Disease 2019: A Clinical Review. Eur. Rev. Med. Pharmacol. Sci..

[2] Bourgonje A.R., Abdulle A.E., Timens W., Hillebrands J., Bourgonje A.R., Abdulle A.E., Timens W., Hillebrands J. (2019). Angiotensin-converting enzyme 2 (ACE2), SARS-CoV-2 and the pathophysiology of coronavirus disease 2019 (COVID-19). J. Pathol..

[3] Driggin E., Madhavan M.V., Bikdeli B., Chuich T., Laracy J., Biondi-Zoccai G., Brown T.S., Der Nigoghossian C., Zidar D.A., Haythe J. (2020). Cardiovascular Considerations for Patients, Health Care Workers, and Health Systems during the COVID-19 Pandemic. J. Am. Coll. Cardiol..

[4] Nie S., Yu M., Xie T., Yang F., Wang H., Wang Z., Li M., Gao X., Lv B., Wang S. (2020). Cardiac Troponin I Is an Independent Predictor for Mortality in Hospitalized Patients with Coronavirus Disease 2019. Circulation.

[5] Shi S., Qin M., Shen B., Cai Y., Liu T., Yang F., Gong W., Liu X., Liang J., Zhao Q. (2020). Association of Cardiac Injury with Mortality in Hospitalized Patients with COVID-19 in Wuhan, China. JAMA Cardiol..

[6] Toraih E.A., Elshazli R.M., Hussein M.H., Elgaml A., Amin M.N., El-Mowafy M., El-Mesery M., Ellythy A., Duchesne J., Killackey M.T. (2020). Association of Cardiac Biomarkers and Comorbidities with Increased Mortality, Severity, and Cardiac Injury in COVID-19 Patients: A Meta-regression and Decision Tree Analysis. J. Med. Virol..

[7] Bavishi C., Bonow R.O., Trivedi V., Abbott J.D., Messerli F.H., Bhatt D.L. (2020). Acute Myocardial Injury in Patients Hospitalized with COVID-19 Infection: A Review. Prog. Cardiovasc. Dis..

[8] Thygesen K., Alpert J.S., Jaffe A.S., Chaitman B.R., Bax J.J., Morrow D.A., White H.D., Mickley H., Crea F., Van De Werf F. (2019). Fourth Universal Definition of Myocardial Infarction (2018). Eur. Heart J..

[9] Charlson M., Szatrowsky T.P., Peterson J., Gold J. (1994). Validation of a Combined Comorbidity Index. Clin. Epidemiol..

[10] Thachil J., Tang N., Gando S., Levi M., Clark C., Iba T., Falanga A., Cattaneo M. (2020). ISTH Interim Guidance on Recognition and Management of Coagulopathy in COVID-19. J. Thromb. Haemost..

[11] Mehran R., Rao S.V., Bhatt D.L., Gibson C.M., Caixeta A., Eikelboom J., Kaul S., Wiviott S.D., Menon V., Nikolsky E. (2011). Standardized Bleeding Definitions for Cardiovascular Clinical Trials: A Consensus Report from the Bleeding Academic Research Consortium. Circulation.

[12] Gebhard C., Regitz-zagrosek V., Neuhauser H.K., Morgan R., Klein S.L. (2020). Impact of Sex and Gender on COVID-19 Outcomes in Europe. Biol. Sex Differ..

[13] Denmark I. (2020). Charlson Comorbidity Index Score and Risk of Severe Outcome and Death in Danish COVID-19 Patients. J. Gen. Intern. Med..

[14] Wan D., Hu B., Hu C., Zhu F., Liu X., Zhang J. (2020). Clinical Characteristics of 138 Hospitalized Patients With 2019 Novel Coronavirus–Infected Pneumonia in Wuhan, China. JAMA Cardiol..

[15] Huang C., Wang Y., Li X., Ren L., Zhao J., Hu Y., Zhang L., Fan G., Xu J., Gu X. (2020). Clinical Features of Patients Infected with 2019 Novel Coronavirus in Wuhan, China. Lancet.

[16] Tang N., Bai H., Chen X., Gong J., Li D., Sun Z. (2020). Anticoagulant Treatment Is Associated with Decreased Mortality in Severe Coronavirus Disease 2019 Patients with Coagulopathy. J. Thromb. Haemost..

[17] Guo T., Fan Y., Chen M., Wu X., Zhang L., He T., Wang H., Wan J., Wang X., Lu Z. (2020). Cardiovascular Implications of Fatal Outcomes of Patients with Coronavirus Disease 2019 (COVID-19). JAMA Cardiol..

[18] Chen L., Li X. (2020). The ACE2 Expression in Human Heart Indicates New Potential Mechanism of Heart Injury among Patients Infected with SARS-CoV-2. Cardiovasc. Res..

[19] Varga Z., Flammer A.J., Steiger P., Haberecker M., Andermatt R., Zinkernagel A.S., Mehra M.R., Schuepbach R.A., Ruschitzka F., Moch H. (2020). Endothelial Cell Infection and Endotheliitis in COVID-19. Lancet.

[20] García L.F. (2020). Immune Response, Inflammation, and the Clinical Spectrum of COVID-19. Front. Immunol..

[21] Merad M., Martin J.C. (2020). Pathological Inflammation in Patients with COVID-19: A Key Role for Monocytes and Macrophages. Nat. Rev. Immunol..

[22] Franchini M., Marano G., Cruciani M., Mengoli C., Pati I., Masiello F., Veropalumbo E., Pupella S., Vaglio S. (2020). COVID-19-Associated Coagulopathy. Diagnosis.

[23] Ortega-Paz L., Capodanno D., Montalescot G., Angiolillo D.J. (2020). COVID-19 Associated Thrombosis and Coagulopathy: Review of the Pathophysiology and Implications for Antithrombotic Management. J. Am. Heart Assoc..

[24] Becker R.C. (2020). COVID-19 Update: Covid-19—Associated Coagulopathy. J. Thromb. Thrombolysis.

[25] Luan Y., Liu Y., Liu X., Yu B., Chen R., Peng M., Ren D. (2020). Coronavirus Disease 2019 (COVID-19) Associated Coagulopathy and Its Impact on Outcomes in Shenzhen, China: A Retrospective Cohort Study. Thromb. Res..

[26] Miesbach W., Makris M. (2020). COVID-19: Coagulopathy, Risk of Thrombosis, and the Rationale for Anticoagulation. Clin. Appl. Thromb. Hemost..

[27] Ayerbe L., Risco C., Ayis S. (2020). The Association between Treatment with Heparin and Survival in Patients with Covid-19. J. Thromb. Thrombolysis.

[28] Paranjpe I., Fuster V., Lala A., Russak A.J., Glicksberg B.S., Levin M., Charney A., Narula J., Fayad Z.A., Bagiella E. (2020). Association of Treatment Dose In-Hospital Survival Among Hospitalized Patients With COVID-19. J. Am. Coll. Cardiol..

[29] Lemos A.C.B., do Espírito Santo D.A., Salvetti M.C., Gilio R.N., Agra L.B., Pazin-Filho A., Miranda C.H. (2020). Therapeutic versus Prophylactic Anticoagulation for Severe COVID-19: A Randomized Phase II Clinical Trial (HESACOVID). Thromb. Res..

[30] Khan I.H., Savarimuthu S., Leung M.S.T., Harky A. (2020). The Need to Manage the Risk of Thromboembolism in COVID-19 Patients. J. Vasc. Surg..

[31] Atallah B., Mallah S.I., AlMahmeed W. (2020). Anticoagulation in COVID-19. Eur. Heart J. Cardiovasc. Pharmacother..

