# Supplementary material for: Myocardial Injury in COVID-19 Patients: Association with Inflammation, Coagulopathy and In-Hospital Prognosis
Source: J Clin Med. 2021 May 13;10(10):2096. doi: 10.3390/jcm10102096 (PMC8152726; doi:10.3390/jcm10102096)
Supplement: Supplementary file 1 [file jcm-10-02096-s001.zip › Table S1.pdf]

Table S1. Baseline medication.

	Myocardial injury		
	**With (n=72)**	**Without (n=259)**	**P-value**
Aspirin, n (%)	28 (38.9)	34 (13.1)	0.001
P2Y12, n (%)	6 (8.3)	5 (1.9)	0.016
ACEI or ARB, n (%)	41 (56.9)	75 (30.0)	0.001
ACEI	15 (20.8)	46 (17.8)	0.606
ARBs	26 (36.1)	29 (11.2)	0.001
Statins, n (%)	30 (41.7)	55 (21.2)	0.001
Beta blockers, n (%)	30 (41.7)	37 (14.3)	0.001
Calcium channel antagonist, n (%)	19 (26.4)	30 (11.6)	0.004
Loop diuretics, n (%)	19 (26.4)	18 (6.9)	0.001
MRA, n (%)	5 (6.9)	5 (1.9)	0.044
Proton pump inhibitor, n (%)	32 (44.4)	67 (25.9)	0.003
Oral hypoglycemic, n (%)	16 (22.2)	30 (11.6)	0.032
Insulin, n (%)	14 (19.4)	9 (3.5)	0.001
Any anticoagulants, n (%)	10 (13.9)	16 (6.2)	0.045
Aspirin, n (%)	28 (38.9)	34 (13.1)	0.001
P2Y12, n (%)	6 (8.3)	5 (1.9)	0.016
ACEI or ARB, n (%)	41 (56.9)	75 (30.0)	0.001
ACEI	15 (20.8)	46 (17.8)	0.606
ARBs	26 (36.1)	29 (11.2)	0.001
Statins, n (%)	30 (41.7)	55 (21.2)	0.001
Beta blockers, n (%)	30 (41.7)	37 (14.3)	0.001
Calcium channel antagonist, n (%)	19 (26.4)	30 (11.6)	0.004
Loop diuretics, n (%)	19 (26.4)	18 (6.9)	0.001

ACEI, angiotensin converting-enzyme inhibitors; ARB, angiotensin II receptor blockers; MRA, mineralocorticoid receptor antagonists.
