# Supplementary material for: Myocardial Injury in COVID-19 Patients: Association with Inflammation, Coagulopathy and In-Hospital Prognosis
Source: J Clin Med. 2021 May 13;10(10):2096. doi: 10.3390/jcm10102096 (PMC8152726; doi:10.3390/jcm10102096)
Supplement: Supplementary file 1 [file jcm-10-02096-s001.zip › Table S2.pdf]

Table S2. Symptoms.

	Myocardial injury		
	**With (n=72)**	**Without (n=259)**	**P-value**
Fever, n (%)	61 (92.4)	228 (90.8)	0.811
Dyspnea, n (%)	44 (66.7)	142 (56.6)	0.161
Cough	44 (66.7)	188 (74.9)	0.043
Non-productive, n (%)	32 (48.5)	164 (65.3)	-
Productive, n (%)	12 (18.2)	24 (9.6)	-
Diarrhea, n (%)	12 (18.2)	53 (21.1)	0.732
