# Supplementary material for: Myocardial Injury in COVID-19 Patients: Association with Inflammation, Coagulopathy and In-Hospital Prognosis
Source: J Clin Med. 2021 May 13;10(10):2096. doi: 10.3390/jcm10102096 (PMC8152726; doi:10.3390/jcm10102096)
Supplement: Supplementary file 1 [file jcm-10-02096-s001.zip › Table S3.pdf]

Table S3. In-hospital treatment.

Drugs	Myocardial injury With (n=72)	Myocardial injury Without (n=259)	P-value
Aspirin, n (%)	30 (43.5)	31 (12.3)	0.001
P2Y12, n (%)	6 (8.7)	5 (2.0)	0.015
ACEI or ARB, n (%)	16 (23.1)	43 (17.1)	0.150
ACEI	9 (13.0)	28 (11.1)	
ARB	7 (10.1)	15 (6.0)	
Statins, n (%)	20 (29.0)	38 (15.1)	0.013
Beta blockers, n (%)	27 (39.1)	35 (13.9)	0.001
Calcium channel antagonist, n (%)	28 (40.6)	65 (25.8)	0.024
Loop diuretics, n (%)	44 (63.8)	58 (23.0)	0.001
MRA, n (%)	4 (5.8)	5 (2.0)	0.105
Lopinavir/Ritonavir, n (%)	51 (72.9)	221 (85.3)	0.020
Hydroxychloroquine, n (%)	66 (94.3)	245 (94.6)	0.920
Azithromycin, n (%)	59 (84.3)	235 (90.7)	0.129
MAB, n (%)	31 (43.1)	124 (47.9)	0.277
Tocilizumab	20 (27.8)	94 (36.3)	
Canakinumab	7 (9.7)	19 (7.3)	
Siltuximab	4 (5.6)	11 (4.2)	
Corticosteroid, n (%)	45 (64.3)	123 (47.5)	0.015
Remdesivir, n (%)	1 (1.4)	20 (7.7)	0.057
NSAIDs, n (%)	3 (4.3)	19 (7.3)	0.589
Paracetamol, n (%)	59 (84.3)	230 (88.8)	0.307

ACEI, angiotensin converting-enzyme inhibitors; ARB, angiotensin II receptor blockers; MRA, mineralocorticoid receptor antagonists; NSAIDs, non-steroidal anti-inflammatory drugs; MAB, monoclonal antibodies.
