# Supplementary material for: Myocardial Injury in COVID-19 Patients: Association with Inflammation, Coagulopathy and In-Hospital Prognosis
Source: J Clin Med. 2021 May 13;10(10):2096. doi: 10.3390/jcm10102096 (PMC8152726; doi:10.3390/jcm10102096)
Supplement: Supplementary file 1 [file jcm-10-02096-s001.zip › Table S4.pdf]

Table S4. Myocardial infarction during hospitalization.

ACS	Age (years)	Sex	CCI	CAC	CRP	ACS day*	Myocardial injury previous to ACS**	Coronary angiography	Treatment	Antiplatelets	Survival
STEMI	74	W	4	2	12.58	8	Yes	No	Thrombolysis	ASA, P2Y12 inhibitor	Death
NSTEMI	67	M	3	4	25.07	7	Yes	Yes	CABG	AAS	Discharge
NSTEMI	80	W	5	3	25.55	2	No	No	Medical treatment	ASA	Death
NSTEMI	72	M	6	1	29.84	9	Yes	No	Medical treatment	ASA	Death
NSTEMI	81	M	4	2	11.12	1	Unknown	No	Medical treatment	None	Death
NSTEMI	67	M	2	0	37.08	9	Yes	No	Medical treatment	None	Death
NSTEMI	81	W	5	0	-	2	Yes	No	Medical treatment	ASA	Death
STEMI	59	M	4	1	25.93	7	No	No	Thrombolysis	ASA, P2Y12 inhibitors	Death
NSTEMI	65	M	5	0	25.19	1	Unknown	No	Medical treatment	ASA, P2Y12 inhibitors	Discharge
NSTEMI	59	M	3	2	16.22	3	Yes	No	Medical treatment	ASA	Death

ACS, acute coronary syndrome; ASA, acetylsalicylic acid; CAC, COVID-19 associated coagulopathy; CCI, Charlson Comorbidity Index; CRP, C-reactive protein.
* Day of hospitalization.
